# Corns

**Published:** 1873-05

**Authors:** 


### CORNS.

The latest cure announced by medical authority is to pare the corn every night, and bind on it a rag saturated with castor oil, so as to remain in contact with the corn during the night; or a poultice of peach leaves. The principle of cure should be always borne in mind. Whatever keeps a common hard corn on the toe moist for twenty-four hours, will so soften it that it may be picked out with the thumb-nail. Fatal bleedings have followed the paring of a corn. Soak a corn twenty minutes at a time in quite warm water, night and morning, and in forty-eight hours the most painful corn will cease hurting, and it may be picked out with the finger-nail. Repeat as often as it reappears.

A gentleman who had suffered with corns on the inner-side of the little toes for forty years, experienced perfect relief in two weeks by applying night, and morning, with a soft brush, one drop of the per-chloride of iron.

